# Impact of Quinolone Prophylaxis on Spontaneous Bacterial Peritonitis and Mortality in Cirrhosis Patients: A Systematic Review and Meta‐Analysis of Randomized Controlled Trials

**DOI:** 10.1002/jgh3.70148

**Published:** 2025-04-17

**Authors:** Ajay Malvi, Nipun Verma, Mahalaqua Nazli Khatib, Subbulakshmi Ganesan, Mandeep Kaur, Manish Srivastava, Amit Barwal, G. V. Siva Prasad, Pranchal Rajput, Rukshar Syed, Kamal Kundra, Kratika Sharma, Diptismitha Jena, Frederick Sidney Correa, Abhinav Rathour, Ganesh Bushi, Rachana Mehta, Sanjit Sah, Prakasini Satapathy, Shilpa Gaidhane, Muhammed Shabil, Hashem Abu Serhan

**Affiliations:** ^1^ National Institute of Pharmaceutical Education and Research Guwahati India; ^2^ Department of Hepatology Postgraduate Institute of Medical Education and Research Chandigarh India; ^3^ Division of Evidence Synthesis, Global Consortium of Public Health and Research Datta Meghe Institute of Higher Education Wardha India; ^4^ Department of Chemistry and Biochemistry, School of Sciences JAIN (Deemed to Be University) Bangalore Karnataka India; ^5^ Department of Allied Healthcare and Sciences Vivekananda Global University Jaipur India; ^6^ Department of Endocrinology NIMS University Jaipur India; ^7^ Chandigarh Pharmacy College Chandigarh Group of College Mohali Punjab India; ^8^ Department of Chemistry Raghu Engineering College Visakhapatnam Andhra Pradesh India; ^9^ School of Applied and Life Sciences, Division of Research and Innovation Uttaranchal University Dehradun India; ^10^ IES Institute of Pharmacy IES University Bhopal Madhya Pradesh India; ^11^ New Delhi Institute of Management New Delhi India; ^12^ Department of Emergency, Graphic Era Institute of Medical Sciences Graphic Era (Deemed to Be University) Clement Town Dehradun India; ^13^ Center for Global Health Research, Saveetha Medical College and Hospital, Saveetha Institute of Medical and Technical Sciences Saveetha University Chennai India; ^14^ Centre of Research Impact and Outcome Chitkara University Rajpura Punjab India; ^15^ Chitkara Centre for Research and Development Chitkara University Chitkara Himachal Pradesh India; ^16^ School of Pharmaceutical Sciences Lovely Professional University Phagwara India; ^17^ Clinical Microbiology, RDC Manav Rachna International Institute of Research and Studies Faridabad Haryana India; ^18^ SR Sanjeevani Hospital, Kalyanpur Siraha Nepal; ^19^ Department of Paediatrics, Dr. D. Y. Patil Medical College Hospital and Research Centre Dr. D. Y. Patil Vidyapeeth (Deemed‐To‐Be‐University) Pune Maharashtra India; ^20^ Department of Medicine Korea Universtiy Seoul South Korea; ^21^ University Center for Research and Development Chandigarh University Mohali Punjab India; ^22^ Medical Laboratories Techniques Department AL‐Mustaqbal University Hillah Babil Iraq; ^23^ One Health Centre (COHERD), Jawaharlal Nehru Medical College Datta Meghe Institute of Higher Education Wardha India; ^24^ Noida Institute of Engineering and Technology (Pharmacy Institute) Greater Noida India; ^25^ Department of Ophthalmology, Hamad Medical Corporation Doha Qatar

**Keywords:** cirrhosis, infections, peritonitis, prophylaxis, quinolone

## Abstract

**Background:**

Cirrhosis is a major global health concern due to its progressive nature and high risk of complications, including spontaneous bacterial peritonitis (SBP), which significantly increases mortality. Quinolone antibiotics, especially norfloxacin, are commonly used for SBP prophylaxis in high‐risk cirrhotic patients, but the long‐term impact on overall mortality remains uncertain. The purpose of this meta‐analysis and systematic review is to evaluate how quinolone prophylaxis affects the SBP incidence, mortality, and non‐SBP infections in cirrhosis patients.

**Methods:**

A comprehensive search of Web of Science, Embase, and PubMed identified research evaluating quinolone prophylaxis on the risk of spontaneous bacterial peritonitis (SBP) and mortality in cirrhotic patients. Inclusion criteria included randomized controlled trials reporting risk ratios for patients on quinolone prophylaxis versus controls. A random‐effects meta‐analysis pooled the results, with heterogeneity assessed by the I^2^ statistic. Sensitivity analyses were performed for robustness.

**Results:**

The search screened 1754 items and identified 6 relevant studies. Quinolone prophylaxis was associated with a significantly lower risk of spontaneous bacterial peritonitis (SBP), non‐SBP infections, and mortality in cirrhotic patients, with a pooled relative risk (RR) for SBP of 0.47 (95% CI: 0.22–1.01), for non‐SBP infections of 0.79 (95% CI: 0.66–0.94), and for mortality of 0.67 (95% CI: 0.52–0.86). Sensitivity analysis confirmed the robustness of these findings.

**Conclusion:**

This meta‐analysis reveals that quinolone prophylaxis significantly lowers the risk of spontaneous bacterial peritonitis (SBP), other infections, and mortality in high‐risk cirrhotic patients. The results support incorporating quinolone prophylaxis in cirrhosis management to improve outcomes, with future studies needed to refine treatment duration and patient‐specific strategies.

## 1 Introduction

Cirrhosis is a progressive liver disease affecting over 1.5 billion people worldwide [1], characterized by extensive scarring and impaired liver function, and it often leads to severe complications and increased mortality [2]. One of the most critical complications of cirrhosis is spontaneous bacterial peritonitis (SBP), an infection of the ascitic fluid that significantly worsens patient outcomes and accelerates liver deterioration [3]. SBP is caused by the translocation of gut‐derived bacteria, primarily *Escherichia coli* , *Klebsiella pneumoniae*, and Enterococcus species. A compromised gut barrier, immune dysfunction, and altered gut microbiota in cirrhotic patients facilitate bacterial migration into the bloodstream and peritoneal cavity, leading to SBP. In patients with cirrhosis, once SBP is established, it triggers a systemic inflammatory response, often progressing to sepsis, renal failure, and further worsening of liver function, exacerbating complications such as ascites, variceal bleeding, and hepatic encephalopathy. SBP occurs in approximately 10%–30% of cirrhotic patients with ascites, with mortality rates reaching as high as 30% following diagnosis [4], emphasizing the urgent need for effective preventive strategies to improve patient survival and reduce the risk of this serious condition.

Quinolone antibiotics, particularly norfloxacin, are widely recommended as a prophylactic measure for high‐risk cirrhotic patients to lower the risk of SBP and potentially reduce related mortality [5]. These antibiotics are thought to prevent SBP by inhibiting bacterial translocation from the gut to the bloodstream and ascitic fluid, thus addressing a major source of infection [5]. However, despite their widespread use, the long‐term impact of quinolone prophylaxis on both SBP prevention and overall mortality remains a matter of debate. Primary prophylaxis refers to the use of antibiotics to prevent the first occurrence of a disease in high‐risk patients. Primary prophylaxis for SBP is recommended in cirrhotic patients with Child‐Pugh class B or C cirrhosis (Child‐Pugh score > 9) who have ascitic fluid protein levels less than 15 g/L, as these patients are at high risk for developing SBP. In contrast, secondary prophylaxis aims to prevent recurrence of SBP in patients who have previously experienced an episode. Recent studies suggest that **rifaximin**, a gut‐selective, non‐absorbable antibiotic, may be equally effective for SBP prophylaxis while **reducing the risk of bacterial resistance** [6]. Additionally, **trimethoprim‐sulfamethoxazole (TMP‐SMX)** is another alternative used in some regions, particularly in the United States, for SBP prophylaxis [7, 8]. These emerging strategies highlight the evolving landscape of infection prevention in cirrhosis. Research findings on the efficacy and safety of quinolone prophylaxis have been mixed; some studies suggest that it effectively reduces SBP risk and improves survival outcomes [9], while other studies suggest that quinolones may have little effect on mortality, and any observed benefits are mostly due to managing other health issues alongside cirrhosis, rather than the use of quinolones alone [10]. Additionally, concerns about the potential development of multi‐drug resistance (MDR) and fungal infections from prolonged quinolone use raise questions about its long‐term safety and impact on patient outcomes, warranting further investigation.

In recent years, research interest has grown in understanding the broader impact of quinolone prophylaxis on overall survival in cirrhotic patients, beyond simply preventing SBP [11, 12]. While some studies propose that reducing SBP risk indirectly enhances survival rates, evidence on the direct effect of quinolone prophylaxis on mortality remains inconclusive, with variations in outcomes potentially influenced by factors such as study design, patient population, and differing follow‐up durations. Furthermore, the presence of common comorbidities in cirrhotic patients, such as renal impairment and hepatic encephalopathy, may also affect mortality outcomes and complicate the evaluation of quinolone effectiveness [13].

Several mechanisms have been suggested to explain the potential mortality benefits of quinolone prophylaxis in cirrhotic patients. By reducing bacterial translocation, quinolones may decrease the frequency and severity of infections, a major cause of death in this population [14]. Additionally, quinolone prophylaxis may reduce systemic inflammation, which is central to the progression of cirrhosis and mortality risk [15]. Nevertheless, the association between quinolone prophylaxis, SBP prevention, and mortality reduction has not been consistently observed, with some studies attributing improved outcomes to the management of comorbid conditions rather than the effects of quinolone alone [16].

A systematic review and meta‐analysis are needed to consolidate current knowledge on the impact of quinolone prophylaxis on SBP risk and mortality in cirrhotic patients. This review aims to clarify the effectiveness of quinolone prophylaxis in reducing SBP occurrence, assess its impact on survival outcomes, and evaluate potential benefits and limitations. This analysis aimed to give a thorough assessment of quinolone prophylaxis by synthesizing the literature, providing insightful information to advise future research paths and therapeutic decisions.

## 2 Methods

### 2.1 Study Design

This systematic review and meta‐analysis were performed to gather and analyze existing research on the effects of quinolone prophylaxis in reducing the risk of SBP and mortality among patients with cirrhosis. The Preferred Reporting Items for Systematic Reviews and Meta‐Analyses (PRISMA) standards were followed in this study [17] (Table S1) to ensure a thorough and transparent approach. The study protocol was also registered with the International Prospective Register of Systematic Reviews (PROSPERO).

### 2.2 Data Sources and Search Strategy

A comprehensive search was carried out across numerous electronic databases, including Web of Science, PubMed, and EMBASE, to identify studies published up to November 2024, examining the effects of quinolone prophylaxis on the risk of SBP and mortality in cirrhotic patients. The search strategy included a combination of free text keywords using terms such as (“Cirrhosis” OR “Liver Cirrhosis” OR “Cirrhotic”) AND (“Quinolone Prophylaxis” OR “Norfloxacin” OR “Ciprofloxacin”) AND (“Spontaneous Bacterial Peritonitis” OR “SBP”) AND (“Mortality” OR “Survival”). No restrictions were applied regarding language or article type. The complete search strategy is outlined in Table S2.

### 2.3 Eligibility Criteria

The following criteria were applied to include studies in this review: [1] only randomized controlled trials (RCTs) assessing the impact of quinolone prophylaxis on the risk of SBP and mortality in cirrhotic patients were included; [2] participants were diagnosed with cirrhosis, regardless of etiology, confirmed through clinical, imaging, or histological criteria; [3] studies provided original quantitative data on SBP risk, mortality rates, or both; and [4] studies were published as peer‐reviewed journal articles. The primary intervention of interest was quinolone prophylaxis, with outcomes focused on the occurrence of SBP and mortality rates. Exclusion criteria included non‐English language publications, case reports, conference abstracts, editorials, reviews, and studies focusing exclusively on other antibiotics or prophylactic interventions. Additionally, studies evaluating alternative prophylactic antibiotics (such as TMP‐SMX or rifaximin) or those lacking clear quantitative data on SBP or mortality outcomes were not included.

### 2.4 Study Selection

The titles and abstracts of every study that was found were first evaluated by two impartial reviewers to ascertain eligibility. For final inclusion, the same reviewers retrieved and examined the full texts of papers that were judged pertinent. A third reviewer was consulted if required to reach a consensus after disagreements were settled via conversation. In order to increase the selection process's efficiency, we employed semi‐automated software (Nested‐Knowledge, MN, USA) to help with the screening process and eliminate duplicates.

### 2.5 Data Extraction

Data were extracted using a standardized form to ensure consistency. Information collected included key study characteristics (such as author, publication year, and country), sample size, study design, participant demographics (e.g., age and gender), criteria used for diagnosing SBP infections, and data on SBP infection rates and mortality outcomes. The data extraction was carried out separately by two reviewers, and any disagreements were settled by discussion or, if required, by bringing in a third reviewer. The Nested‐Knowledge software's “tagging” capability was used to speed up the extraction process.

### 2.6 Risk of Bias Assessment

The risk of bias in the included RCTs was assessed using the Revised Cochrane risk‐of‐bias tool for randomized trials (RoB 2). The tool evaluates bias across five domains: randomization process, deviations from intended interventions, missing outcome data, measurement of the outcome, and selection of the reported result. Each study was rated as having a “low,” “high,” or “some concerns” risk of bias. The assessment was performed independently by two reviewers, with disagreements resolved through discussion.

### 2.7 Data Synthesis and Statistical Analysis

A random‐effects meta‐analysis was done to investigate the influence of quinolone prophylaxis on the risk of SBP and mortality among patients with cirrhosis, allowing for heterogeneity among trials. The I^2^ statistic was used to quantify heterogeneity; values greater than 50% indicated significant heterogeneity. The pooled effect of quinolone prophylaxis on SBP risk and mortality was estimated using this random‐effects model. Statistical significance was determined at a *p*‐value threshold of 0.05 for all analyses. All statistical procedures were performed using R software (version 4.4).

## 3 Results

### 3.1 Literature Search

**FIGURE 1:**
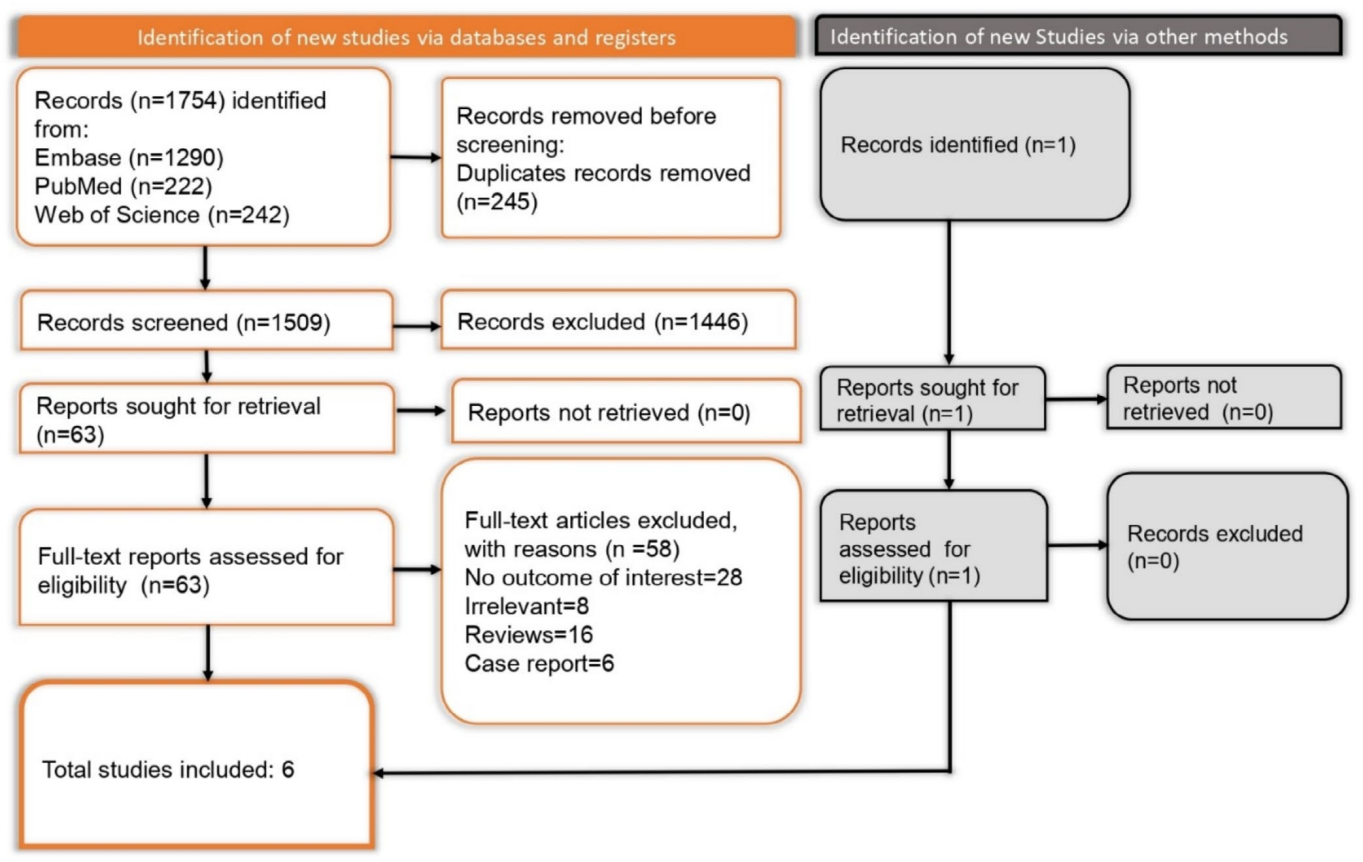
PRISMA flowchart depicting article selection and screening process.

A systematic search across Embase, PubMed, and Web of Science yielded 1754 records in total, with 1290 records from Embase, 222 from PubMed, and 242 from Web of Science. After removing 245 duplicate entries, 1509 unique records were available for screening. Titles and abstracts were initially reviewed, resulting in the exclusion of 1446 records that did not satisfy the inclusion criteria. This process left 63 articles for full‐text evaluation. Upon detailed assessment, 58 articles were excluded due to the following reasons: 28 did not report relevant outcomes, 16 were review articles, 8 were outside the study's scope, and 6 were case reports. Ultimately, 6 studies [10, 18, 19, 20, 21, 22] fulfilled all eligibility criteria and were included in the systematic review (Figure 1).

### 3.2 Characteristics of Included Studies

**FIGURE 2:**
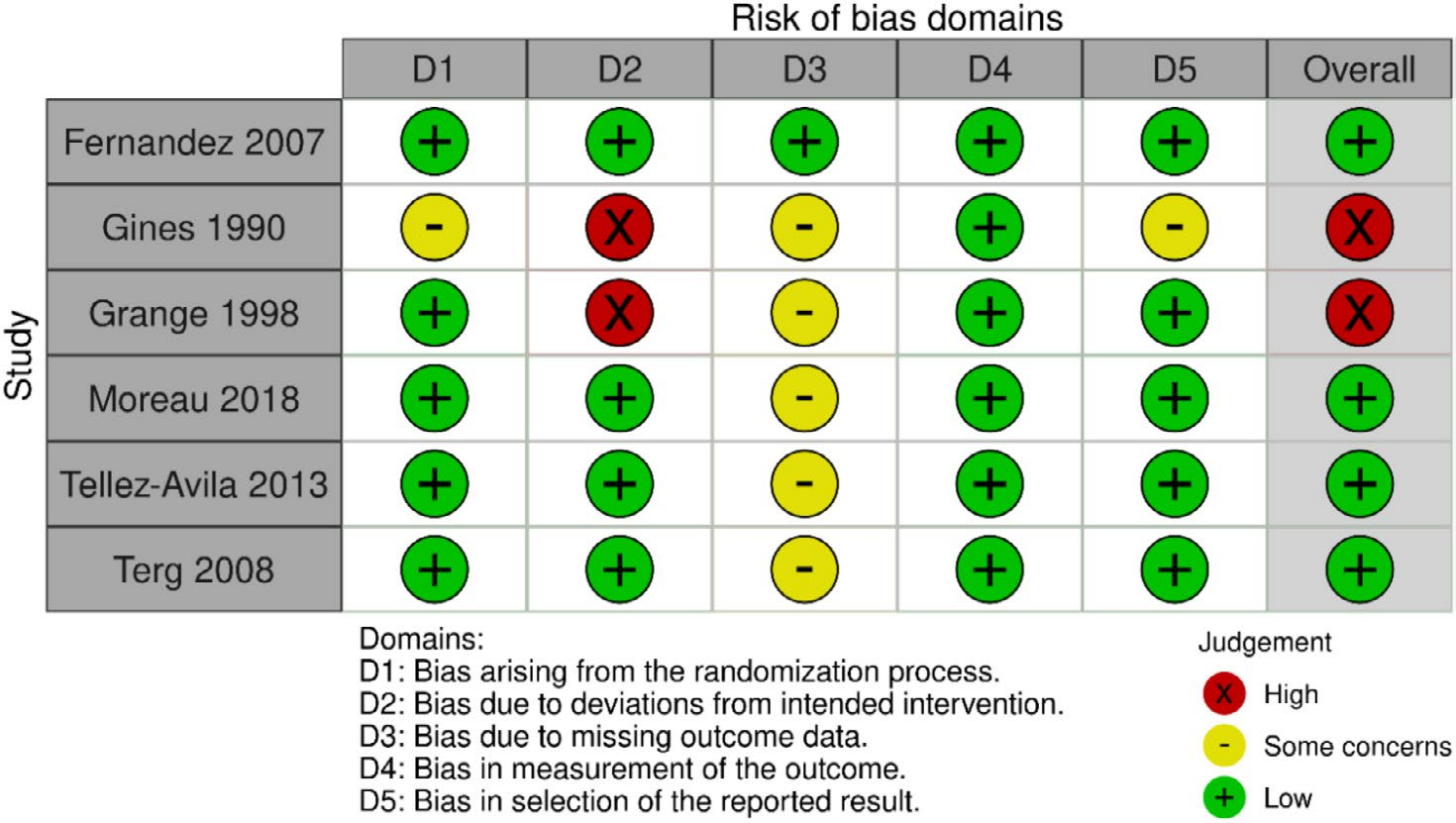
Risk of bias assessment.

The included studies comprised six randomized controlled trials conducted in diverse countries, including Spain, France, Mexico, and Argentina (Table 1). Follow‐up periods ranged from six months to one year, with participants primarily being middle‐aged to older adults with cirrhosis at high risk for SBP due to factors like advanced liver failure or renal impairment. Quinolone prophylaxis, typically norfloxacin at 400 mg/day or ciprofloxacin at 500 mg/day, was compared against placebo. Baseline characteristics of the included patients are summarized in Table 2. The studies reported key clinical parameters, including serum bilirubin levels, Child‐Pugh scores, blood urea nitrogen (BUN) levels, and ascitic fluid protein concentrations, which serve as important indicators of liver function and disease severity. The overall patient population had moderate to severe liver disease, as reflected by variations in bilirubin levels, Child‐Pugh scores, and ascitic fluid protein levels, which are established risk factors for SBP. Sample sizes and statistical power varied across studies, enabling analysis of diverse populations. SBP diagnosis followed standardized criteria, including a polymorphonuclear (PMN) cell count above 250/mm^3^ in ascitic fluid, and several studies reported the Model for End‐Stage Liver Disease (MELD) score to assess liver severity. The risk of bias assessment is presented in Figure 2.

**TABLE 1:** Characteristics of included studies.

Study	Country	Study design	Follow up	Population characteristics	Age (Intervention)	Intervention:Control	Sample size (Intervention:Control)	Male	Dose (Intervention: Control)	SBP infection (Intervention:Control)	Mortality (Intervention:Control)	Diagnostic criteria (SBP)	Meld score
Fernandez 2007 [18]	Spain	Randomized controlled trial	1 year	Patients with cirrhosis and low protein ascitic levels with advanced liver failure or impaired renal function	Intervention = 62 (SD = 11), Control = 61 (SD = 12)	Norfloxacin:Placebo	35:33	Norfloxacin—63% Placebo—67%	400 mg/day:NA	2:10	10:13	NA	Intervention = 16.7 (SD = 3.0), Control = 18.1 (SD = 3.7)
GINES 1990 [19]	Spain	Double‐blind, multicenter, placebo‐ controlled trial	1 year	80 cirrhotic patients	Intervention = 59 (SD = 1), Control = 56 (SD = 2)	Norfloxacin:Placebo	40:40	Norfloxacin—70% Placebo—65%	400 mg/day:NA	5:14	7/10	Abdominal pain/fever, ascitic fluid PMN > 350/mm^3^, and no clinical or imaging signs of secondary peritonitis.	NA
Grange 1998 [20]	France	Multicenter double‐blind, placebo‐con‐trolled randomized trial	6 months	Cirrhotic patients admitted to hospital with low‐protein ascites	Intervention = 55 (35–70), Control = 55 (31–70)	Norfloxacin:Placebo	53:54	Norfloxacin—68% Placebo—59%	400 mg/day:NA	3:3	8/10	Polymorphonuclear cell (PMN) count greater than 250/ml	NA
Moreau 2018 [10]	France	Prospective, multicentre, double‐blind, randomized, parallel‐group, placebo‐controlled, phase 3 trial	12 months	Patients older than 18 years, had Child‐Pugh class C cirrhosis	Intervention = 55.2 (SD = 8.5), Control = 56 (SD = 9.5)	Norfloxacin:Placebo	144:147	Norfloxacin—65% Placebo—74%	1 tablet of 400 mg daily:1 tablet of 400 mg daily	10:17	19:27	NA	Intervention = 21.4 (SD = 5.0), Control = 21.0 (SD = 5.3)
Tellez‐Avila 2013 [21]	Mexico	Randomized, double‐blind placebo‐con‐trolled clinical trial	6 months	Patients with cirrhosis of the liver and ascites	Intervention = 56.7 (SD = 13.2), Control = 56.3 (SD = 11.7)	Ciprofloxacin:Placebo	49:46	Ciprofloxacin—31% Placebo—48%	500 mg/day:500 mg/day	2:0	1/3	> 250 PMN/mm3 ascites	Intervention = 12.9 (SD = 3.8), Control = 12.4 (SD = 2.5)
Terg 2008 [22]	Argentina	Multicenter, randomized, doubleblind, and placebo‐controlled tiral	1 year	Patients with cirrhosis and ascites	Intervention = 56 (SD = 10), Control = 58 (SD = 11)	Ciprofloxacin:Placebo	50:50	NA	500 mg/day:NA	2:7	6/14	Ascitic fluid PMN count > 250/mm^3^ and no intra‐abdominal infection source.	NA

Abbreviations: SBP: Spontaneous Bacterial Peritonitis, PMN: Polymorphonuclear (cell count), MELD Score: Model for End‐Stage Liver Disease Score, SD: Standard Deviation, NA: Not Available, mg/day: Milligrams per day.

**TABLE 2:** Baseline characteristics of the included participants.

Study	Intervention:Control	Serum bilirubin level, mg/Dl (Intervention: Control)	Child–Pugh score (Intervention: Control)	Blood urea nitrogen level, mg/dL (Intervention:Control)	Ascitic fluid protein level, g/L (Intervention:Control)
Fernandez 2007 [18]	Norfloxacin:Placebo	Mean = 3.5 (SD = 2.3):Mean = 4.4 (SD = 4.6)	Mean = 9.9 (SD = 1.5): 10.4 (SD = 1.5)	Mean = 27 (SD = 17):Mean = 29 (SD = 1)	Mean = 9 (SD = 4): Mean = 9 (SD = 3)
GINES 1990 [19]	Norfloxacin:Placebo	Mean = 2.8 (SD = 0.3): Mean = 2.8 (SD = 0.3)	NA	Mean = 25 (SD = 2): Mean = 22 (SD = 2)	1.143055556
Grange 1998 [20]	Norfloxacin:Placebo	Mean = 51.4 (SD = 8.3): Mean = 38.3 (SD = 5.6)	NA	NA	Mean = 9.3 (SD = 2.9): Mean = 10.4 (SD = 2.8)
Moreau 2018 [10]	Norfloxacin:Placebo	Mean = 7.7 (SD = 6.7): Mean = 8.0 (SD = 7.1)	Mean = 11.4 (SD = 1.1): Mean = 11.2 (SD = 1.0)	NA	Mean = 13.0 (SD = 6.6):Mean = 12.3 (SD = 6.8)
Tellez‐Avila 2013 [21]	Ciprofloxacin:Placebo	Mean = 2.2 (sd = 1.3): Mean = 2.08 (sd = 1.2)	Mean = 8.1 (SD = 1.6): Mean = 8.0 (SD = 1.8)	NA	NA
Terg 2008 [22]	Ciprofloxacin:Placebo	Mean = 2.9 (SD = 4.6): Mean = 2.7 (SD = 3.2)	Mean = 8.5 (SD = 1.5): Mean = 8.3 (SD = 1.3)	Mean = 32.2 (SD = 15.6): Mean = 29.7 (SD = 14.6)	Mean = 0.84 (SD = 0.31): Mean = 0.85 (SD = 0.36)

Abbreviations: SD: Standard Deviation, NA: Not Available, mg/dL – milligrams per deciliter; g/L—grams per liter.

### 3.3 Risk of SBP Infections

**FIGURE 3:**
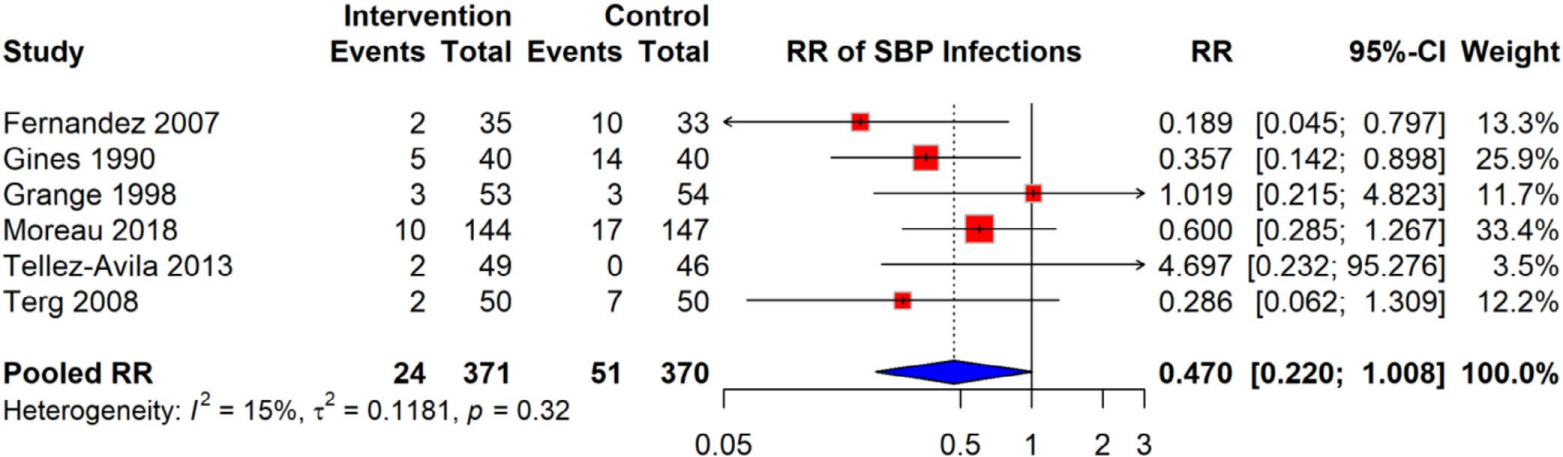
Pooled RR for risk of SBP infection among cirrhosis patients receiving quinolone prophylaxis.

The meta‐analysis of six studies demonstrated that the relative risk (RR) of SBP infections among cirrhosis patients receiving quinolone prophylaxis is 0.470 (95% CI: 0.220–1.008), based on a total of 24 events in the intervention group compared to 51 events in the control group, across 741 participants. The analysis showed low heterogeneity (I^2^ = 15%), indicating minimal variation between studies. RR estimates varied across studies, with some studies reporting RR as low as 0.189 and others as high as 4.697 (Figure 3).

### 3.4 Risk of Mortality

**FIGURE 4:**
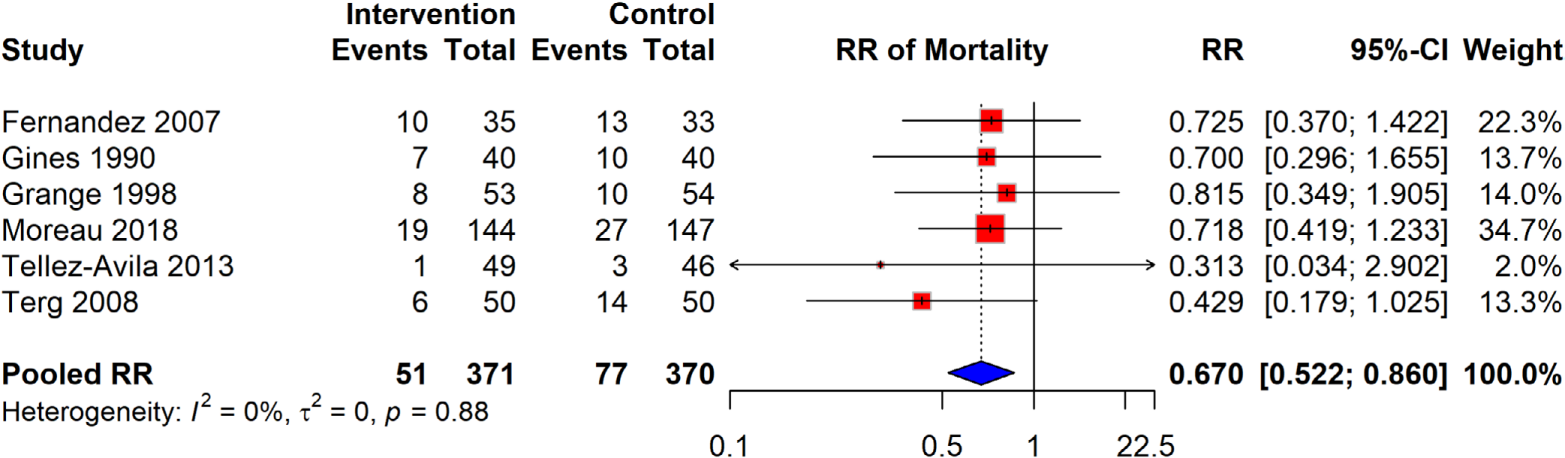
Pooled RR for risk of mortality infection among cirrhosis patients receiving quinolone prophylaxis.

The meta‐analysis of six studies demonstrated that the RR of mortality among cirrhosis patients receiving quinolone prophylaxis was 0.670 (95% CI: 0.522–0.860), based on a total of 51 events in the intervention group compared to 77 events in the control group, across 741 participants. The analysis indicated no significant heterogeneity (I^2^ = 0%), suggesting consistency in effect across the included studies. RR estimates varied across studies, with some studies reporting RR as low as 0.313 and others as high as 0.815 (Figure 4).

### 3.5 Risk of Non‐SBP Infections

**FIGURE 5:**
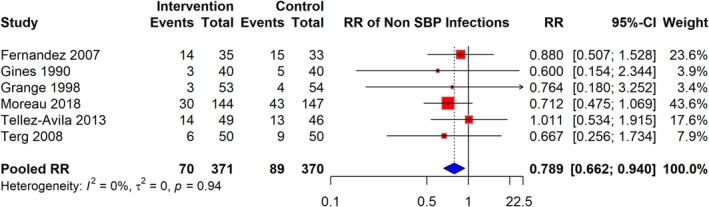
Pooled RR for risk of non‐BP infection among cirrhosis patients receiving quinolone prophylaxis.

The meta‐analysis of six studies demonstrated that the RR of non‐SBP infections among patients receiving quinolone prophylaxis was 0.789 (95% CI: 0.662–0.940), based on a total of 70 events in the intervention group compared to 89 events in the control group, across 741 participants. These non‐SBP infections included urinary tract infections (UTIs), pneumonia, respiratory tract infections (RTIs), and bacteremia. The analysis indicated no significant heterogeneity (I^2^ = 0%), suggesting consistency in effect across the studies. RR estimates varied, with some studies reporting RR as low as 0.600 and others as high as 1.011 (Figure 5).

### 3.6 Subgroup Analysis of Risk of SBP Infection

**FIGURE 6:**
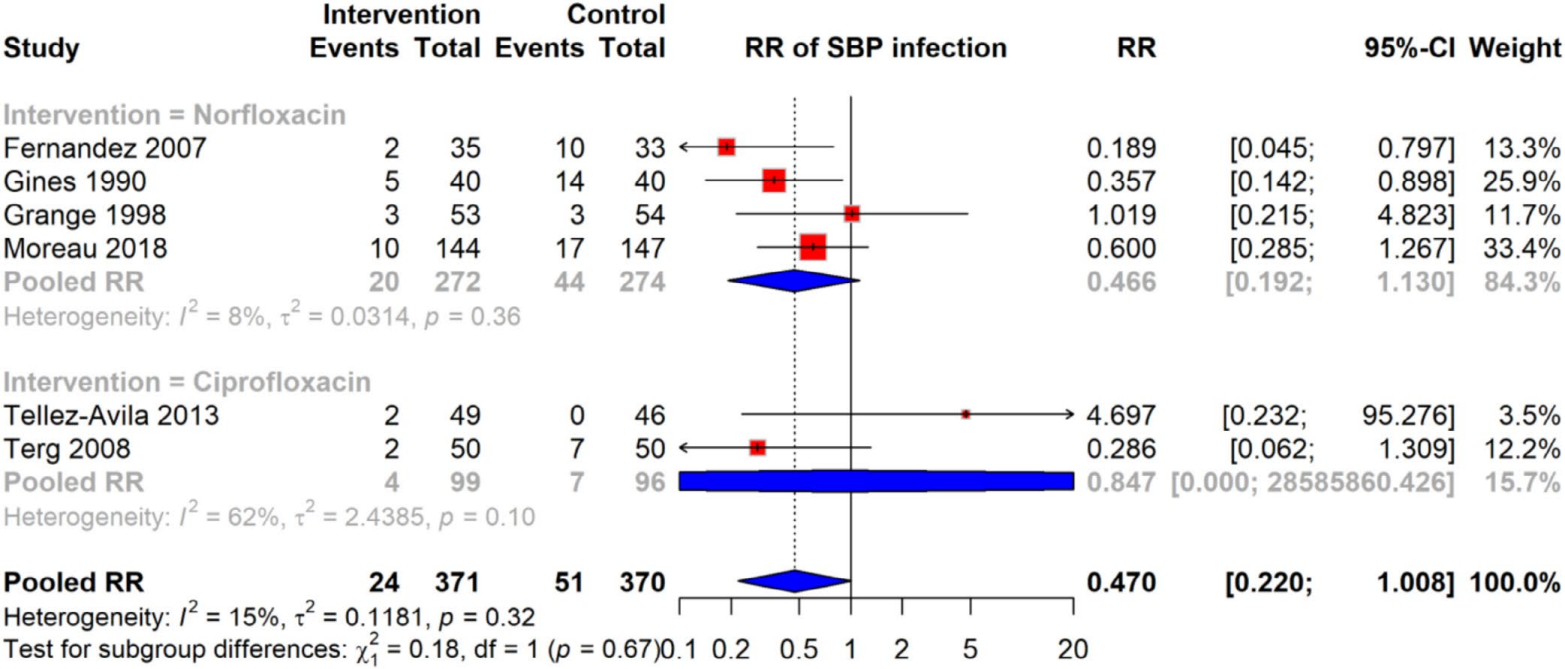
Subgroup analysis of pooled RR showing the risk of SBP infection among cirrhosis patients receiving quinolone prophylaxis.

Subgroup analyses were conducted based on the antibiotic type (Norfloxacin and Ciprofloxacin) to investigate potential sources of heterogeneity in the relationship between antibiotic prophylaxis and the risk of SBP infection. The pooled RR for studies using Norfloxacin was 0.466 (95% CI: 0.192–1.130), while, for studies using Ciprofloxacin, the pooled RR was 0.847 (95% CI: 0.000–2.685). Heterogeneity was low within each subgroup, with I^2^ values of 8% for Norfloxacin and 62% for Ciprofloxacin, indicating some variation among studies using Ciprofloxacin. However, the test for subgroup differences was not statistically significant (*p* = 0.67), suggesting no significant variation between the different antibiotic groups in terms of their effect on SBP infection risk (Figure 6).

### 3.7 Subgroup Analysis of Risk of Mortality

**FIGURE 7:**
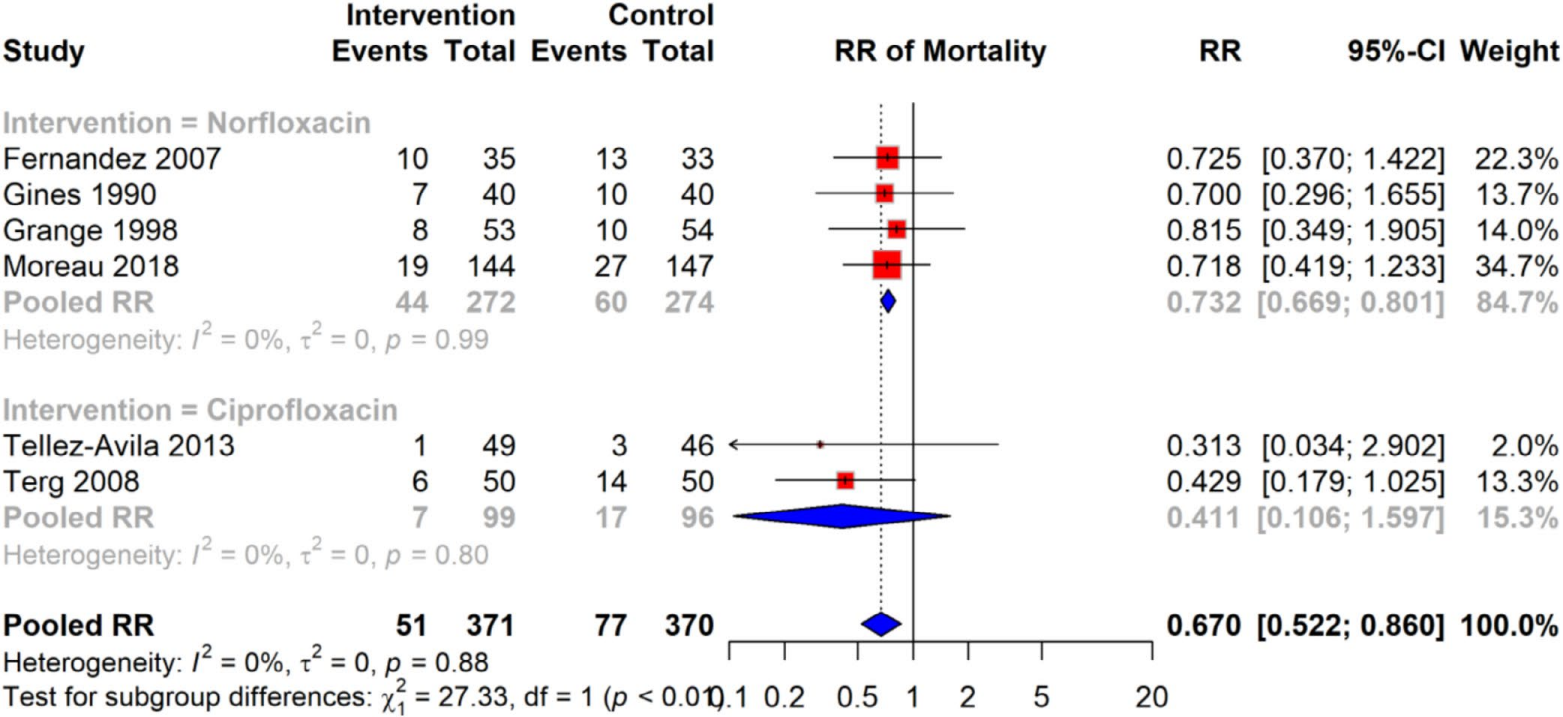
Subgroup analysis of pooled RR showing the risk of mortality among cirrhosis patients receiving quinolone prophylaxis based on the type of quinolone.

Subgroup analyses were performed based on the type of antibiotic used (Norfloxacin and Ciprofloxacin) to explore potential sources of heterogeneity in the relationship between antibiotic prophylaxis and mortality risk. The pooled RR for studies using Norfloxacin was 0.732 (95% CI: 0.689–0.801), while for studies using Ciprofloxacin, the pooled RR was 0.411 (95% CI: 0.106–1.597). Heterogeneity was low across both subgroups, with I^2^ = 0% in both cases, indicating consistent associations regardless of the antibiotic type. The test for subgroup differences was statistically significant (*p* < 0.001), suggesting a notable variation between the effects of Norfloxacin and Ciprofloxacin on mortality risk (Figure 7).

### 3.8 Subgroup Analysis of Risk of Non—SBP Infection

**FIGURE 8:**
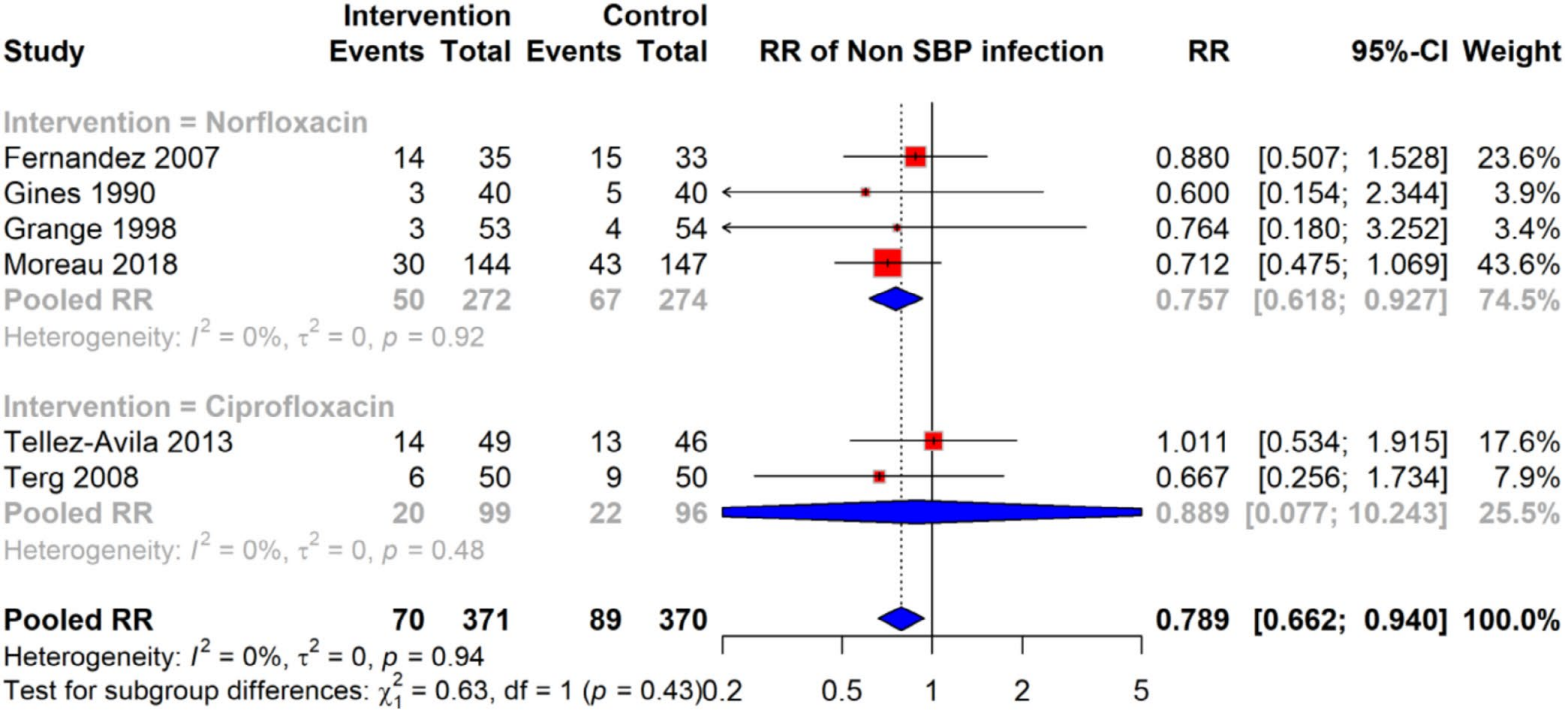
Subgroup analysis of pooled RR showing the risk of non‐BP infection among cirrhosis patients receiving quinolone prophylaxis based on type of quinolone.

Subgroup analyses were performed based on the type of antibiotic used (Norfloxacin and Ciprofloxacin) to explore potential sources of heterogeneity in the association between antibiotic prophylaxis and the risk of non‐SBP infections. The pooled RR for studies using Norfloxacin was 0.757 (95% CI: 0.618–0.927), while for studies using Ciprofloxacin, the pooled RR was 0.889 (95% CI: 0.077–10.243). Heterogeneity was low within both subgroups, with I^2^ = 0% in each case, indicating consistent associations regardless of the antibiotic type. The test for subgroup differences was not statistically significant (*p* = 0.43), suggesting no significant variation between the effects of Norfloxacin and Ciprofloxacin on the risk of non‐SBP infections (Figure 8).

### 3.9 Sensitivity Analysis

A leave‐one‐out sensitivity analysis was conducted to evaluate the robustness of pooled estimates for the outcomes of SBP infection, mortality, and non‐SBP infections among cirrhotic patients receiving quinolone prophylaxis. For SBP infection, pooled RRs ranged from 0.423 to 0.532; for mortality, from 0.649 to 0.718; and for non‐SBP infections, from 0.748 to 0.854. In all cases, excluding any single study did not significantly alter the overall effect size, confirming the stability of the associations between quinolone prophylaxis and reduced risks of SBP infection, mortality, and non‐SBP infections (Figures S1–S3).

## 4 Discussion

The key results from this systematic review and meta‐analysis provide substantial evidence on the efficacy of quinolone prophylaxis in reducing both SBP and mortality in cirrhotic patients, with additional benefits in preventing non‐SBP infections. Specifically, the cumulative RR for SBP, mortality, and non‐SBP infections was 0.470 (95% CI: 0.220–1.008), 0.670 (95% CI: 0.522–0.860), and 0.789 (95% CI: 0.662–0.940), respectively. These findings indicate that cirrhotic patients who receive quinolone prophylaxis have a significantly reduced risk of these infections and mortality. Non‐SBP infections, such as UTIs, pneumonia, RTIs, and bacteremia, were significantly reduced, further highlighting the broad‐spectrum efficacy of quinolone prophylaxis. The risk reduction for SBP was particularly notable, with an estimated 53% decrease in risk, while mortality was reduced by 33%, and non‐SBP infections by 21%. This comprehensive reduction in infection‐related outcomes highlights the utility of quinolone prophylaxis as an effective preventive strategy to improve survival and reduce healthcare burdens in patients with advanced liver disease. The consistent results across these outcomes emphasize the clinical relevance of incorporating quinolone prophylaxis into routine management for high‐risk cirrhotic patients. However, it is important to note that most of the studies included in this meta‐analysis primarily focused on **primary prophylaxis**, aimed at preventing the first occurrence of SBP in cirrhotic patients at risk, such as those with ascites or advanced liver disease. In contrast, **secondary prophylaxis**, which is used to prevent recurrence in patients who have already had an episode of SBP, was only addressed by one study. This distinction in study focus emphasizes the need for further research to evaluate whether quinolone therapy can effectively reduce SBP recurrence in patients who have already experienced an episode of SBP.

The meta‐analysis estimated that quinolone prophylaxis reduces the risk of SBP by approximately 53% (RR = 0.470, 95% CI: 0.220–1.008), based on data from six studies involving over 700 patients. SBP is a severe complication in cirrhotic patients, with mortality rates often ranging as high as 30% [23, 24], making this observed reduction clinically significant, as it may help prevent sudden deteriorations in liver function and reduce the need for emergency medical interventions. Similarly, the analysis showed a 21% reduction in the risk of non‐SBP infections (RR = 0.789, 95% CI: 0.662–0.940), which include common and burdensome infections such as UTIs, pneumonia, RTIs, and bacteremia. These infections contribute significantly to morbidity and healthcare costs in cirrhotic patients. The combined reduction in both SBP and non‐SBP infections highlights the broad‐spectrum potential of quinolone prophylaxis in high‐risk cirrhotic populations, not only in addressing SBP but also in lowering the overall burden of infections, thereby enhancing infection control and potentially improving patient outcomes.

The pooled analysis for mortality demonstrated a RR reduction of approximately 33% (RR = 0.670), reinforcing the potential of quinolone prophylaxis to improve survival outcomes in cirrhosis. This mortality reduction is particularly significant given the high baseline risk of death in cirrhotic patients with ascites and recurrent infections. The consistent reduction in mortality risk across studies highlights quinolone prophylaxis as a valuable addition to standard cirrhosis care, particularly in patients with advanced disease and other risk factors for infection. These findings support existing guidelines recommending the use of quinolone antibiotics, such as norfloxacin, for primary and secondary SBP prophylaxis in high‐risk cirrhotic patients [25].

In comparison to previous meta‐analyses, our study offers novel insights by focusing specifically on the effects of quinolones in cirrhosis management rather than evaluating a broad range of antibiotics [26, 27]. Our findings extend the current understanding by demonstrating that quinolone prophylaxis significantly reduces both SBP and non‐SBP infection risks, contributing to a 33% reduction in mortality. This broader protective effect emphasizes the importance of targeted prophylactic antibiotics in managing cirrhosis, particularly for high‐risk patients with ascites. Observational studies have explored quinolone prophylaxis in cirrhosis management. One study in Argentina and Uruguay found no significant link between norfloxacin use and the development of multidrug‐resistant organisms (MDROs) in 472 cirrhotic patients, supporting its continued use in well‐selected patients [28]. However, another study based on Veterans Affairs (VA) data found that quinolone prophylaxis increased the risk of SBP recurrence and fluoroquinolone resistance, highlighting the need for more targeted, personalized strategies in cirrhosis management to balance the risks of antibiotic resistance [29]. Recent studies have explored rifaximin as an alternative to quinolones, showing comparable efficacy in preventing SBP with the added benefit of lower systemic absorption, which reduces the risk of antibiotic resistance a—major concern with prolonged quinolone use [8]. Its gut‐selectivity makes rifaximin particularly appealing for patients at high risk for quinolone resistance, as it primarily targets intestinal flora with minimal systemic effects. This is especially beneficial for cirrhotic patients, who are more vulnerable to the adverse effects of systemic antibiotics. Rifaximin may offer superior safety in certain populations, particularly in those with advanced liver disease, given its reduced risk of systemic side effects. However, quinolones remain more widely available and may still be an important option, especially in settings where rifaximin is not accessible or affordable. Further studies are needed to evaluate its long‐term safety and efficacy, particularly in relation to resistance development. Despite these promising findings, quinolones remain a valuable tool in SBP prophylaxis, but rifaximin may offer a safer, more sustainable alternative in the future. While quinolone prophylaxis has been shown to reduce SBP recurrence and mortality, some recent studies, such as the RCT from Asia [30], report no mortality benefit and an increased risk of fungal infections with long‐term use. These findings highlight the potential risks of MDR infections and fungal infections, especially in cirrhotic patients with compromised immune systems. Given these concerns, quinolone prophylaxis should be carefully considered for high‐risk patients, emphasizing the need for targeted, personalized strategies to balance the benefits of infection prevention with the risks of prolonged antibiotic use. Concerns regarding antibiotic resistance and the long‐term safety of quinolone prophylaxis remain, highlighting the need for continued research to optimize prophylactic strategies, balance risks, and improve patient outcomes in cirrhosis management.

The substantial heterogeneity observed in studies assessing quinolone prophylaxis for SBP (I^2^ = 15%) reflects variations in patient populations, cirrhosis severity, and clinical protocols across studies. Non‐SBP infections and mortality outcomes exhibited no heterogeneity (I^2^ = 0%), contributing to more consistent findings in these areas. Differences in prophylaxis duration, quinolone dosage, and the presence of coexisting conditions such as renal impairment or hepatic encephalopathy may further influence the variability observed in SBP outcomes. Comorbid conditions, including advanced liver disease or immune‐compromising states, could increase the likelihood of infections among cirrhotic patients, although these factors were not consistently controlled for across studies. According to the subgroup analysis, patients receiving norfloxacin were significantly less likely to develop SBP, with a pooled RR of 0.470 (95% CI: 0.220–1.008) and a corresponding *p*‐value of 0.32. For non‐SBP infections, norfloxacin showed a pooled RR of 0.757 (95% CI: 0.618–0.927) with a *p*‐value of 0.92, while ciprofloxacin showed a slightly higher RR of 0.889 (95% CI: 0.077–10.243) with a *p*‐value of 0.94. In terms of mortality, norfloxacin prophylaxis was associated with a pooled RR of 0.732 (95% CI: 0.689–0.801) and a *p*‐value of 0.99, while ciprofloxacin yielded a pooled RR of 0.411 (95% CI: 0.106–1.597) with a *p*‐value of 0.88. Although the *p*‐values indicate that the differences between norfloxacin and ciprofloxacin are not statistically significant, they highlight potential variations in the efficacy of different quinolones. Sensitivity analyses confirmed the stability of these findings, as the exclusion of individual studies did not substantially alter the pooled estimates. This consistency emphasizes the robustness of quinolone prophylaxis as a preventive strategy, though more research is warranted to ascertain how individual and environmental variables influence the outcomes of quinolone prophylaxis in this high‐risk population.

Several mechanisms have been proposed to explain the observed reduction in SBP, non‐SBP infections, and mortality with quinolone prophylaxis. A major factor is the reduction in bacterial translocation from the gut, a common pathway for infection in cirrhotic patients, by inhibiting bacterial growth and preventing movement into the bloodstream [11]. Additionally, quinolones may mitigate systemic inflammation, a significant contributor to disease progression and infection susceptibility in cirrhosis [31]. Reduced inflammation may not only lower infection risk but also slow liver deterioration, enhancing patient survival [32]. Furthermore, the broad‐spectrum efficacy of quinolones against other infections such as urinary and respiratory tract infections suggests an added benefit, especially in cirrhotic patients with compromised immune systems. These mechanisms, while promising, warrant further investigation to fully understand the pathways through which quinolones contribute to infection prevention and survival in cirrhosis.

The results of this meta‐analysis carry important clinical implications. First, they underscore the potential benefits of incorporating quinolone prophylaxis into routine care for high‐risk cirrhotic patients to reduce both infection risk and mortality. Given the high infection‐related morbidity and mortality in this population, routine prophylaxis could significantly alleviate healthcare burdens by reducing hospitalizations and preventing severe infections. Additionally, these findings suggest the need for a holistic approach in cirrhosis management, where infection prevention through quinolone prophylaxis is combined with strategies to monitor and address potential side effects, such as antibiotic resistance and adverse reactions. However, the long‐term use of quinolones is associated with the development of MDR, which may limit their effectiveness. As antibiotic resistance continues to emerge, personalized prophylaxis strategies are necessary to address the unique risk factors of individual patients. For example, patients with a history of quinolone‐resistant infections, or those living in regions with high resistance rates, may benefit more from alternatives such as rifaximin or TMP‐SMX. A personalized approach, considering comorbidities like renal impairment or hepatic encephalopathy, and regional microbial resistance profiles, will optimize prophylaxis regimens and minimize long‐term complications [33]. Public health policies that support access to prophylactic antibiotics for high‐risk cirrhotic patients could further enhance the impact of these interventions, especially in settings with limited healthcare resources.

Despite offering valuable insights, this meta‐analysis has several limitations that warrant consideration. Substantial heterogeneity in the included studies, especially in the SBP analysis, suggests that specific moderating factors, such as the severity of cirrhosis, type of quinolone used, and the presence of comorbidities, were not fully explored. Future studies should examine the impact of these factors in depth to refine patient selection criteria and prophylaxis protocols. Additionally, while our meta‐analysis focused on randomized controlled trials, the relatively small number of included studies may limit the generalizability of the findings across diverse populations and healthcare settings. The relatively short follow‐up periods (6 months to 1 year) also limit our ability to assess the long‐term efficacy of quinolone prophylaxis, including its effects on liver function and survival over time. One limitation of this meta‐analysis is the predominant focus on primary prophylaxis, with only one study addressing secondary prophylaxis. This highlights a gap in the literature regarding the effectiveness of quinolone therapy in preventing SBP recurrence, emphasizing the need for further research on secondary prophylaxis. Most of the included studies were conducted in a limited range of countries, namely Spain, France, Mexico, and Argentina, which may restrict the applicability of these findings to other regions with different healthcare systems, patient demographics, and clinical practices. Further research, particularly longitudinal studies, is necessary to assess the long‐term effects of quinolone prophylaxis on liver function and survival outcomes. Expanding research across more varied populations and geographic regions will also help ensure that these findings are globally applicable and reflective of broader cirrhotic patient populations.

### 4.1 Conclusion

This meta‐analysis found a significant link between quinolone prophylaxis and reduced risks of SBP, non‐SBP infections, and mortality in cirrhotic patients. These findings emphasize the value of prophylactic antibiotics in high‐risk patients, especially those with advanced liver disease and comorbidities. However, concerns about antibiotic resistance and the long‐term safety of quinolones highlight the need for personalized prophylaxis strategies, considering factors like patient history, regional resistance, and comorbidities. Public health initiatives are essential to raise awareness about infection risks in cirrhosis and the benefits of targeted prophylaxis. Future research should explore alternatives like rifaximin and TMP‐SMX, assessing their long‐term efficacy and safety to optimize prophylactic strategies amid evolving resistance challenges.

## Ethics Statement

The authors have nothing to report.

## Consent

The authors have nothing to report.

## Conflicts of Interest

The authors declare no conflicts of interest.

## Supporting information


**Data S1.** Supplementary Materials

## Data Availability

The data is with the authors and available on request.
